# Correlating Function and Imaging Measures of the Medial Longitudinal Fasciculus

**DOI:** 10.1371/journal.pone.0147863

**Published:** 2016-01-22

**Authors:** Ken Sakaie, Masaya Takahashi, Gina Remington, Xiaofeng Wang, Amy Conger, Darrel Conger, Ivan Dimitrov, Stephen Jones, Ashley Frohman, Teresa Frohman, Koji Sagiyama, Osamu Togao, Robert J. Fox, Elliot Frohman

**Affiliations:** 1 Imaging Institute, Cleveland Clinic, Cleveland, Ohio, United States of America; 2 Advanced Imaging Research Center, University of Texas Southwestern Medical Center at Dallas, Dallas, Texas, United States of America; 3 Department of Neurology and Neurotherapeutics, University of Texas Southwestern Medical Center at Dallas, Dallas, Texas, United States of America; 4 Quantitative Health Sciences, Cleveland Clinic, Cleveland, Ohio, United States of America; 5 Philips Medical Systems, Cleveland, Ohio, United States of America; 6 Mellen Center, Cleveland Clinic, Cleveland, Ohio, United States of America; University of Illinois at Chicago, UNITED STATES

## Abstract

**Objective:**

To test the validity of diffusion tensor imaging (DTI) measures of tissue injury by examining such measures in a white matter structure with well-defined function, the medial longitudinal fasciculus (MLF). Injury to the MLF underlies internuclear ophthalmoparesis (INO).

**Methods:**

40 MS patients with chronic INO and 15 healthy controls were examined under an IRB-approved protocol. Tissue integrity of the MLF was characterized by DTI parameters: longitudinal diffusivity (LD), transverse diffusivity (TD), mean diffusivity (MD) and fractional anisotropy (FA). Severity of INO was quantified by infrared oculography to measure versional disconjugacy index (VDI).

**Results:**

LD was significantly lower in patients than in controls in the medulla-pons region of the MLF (p < 0.03). FA was also lower in patients in the same region (p < 0.0004). LD of the medulla-pons region correlated with VDI (R = -0.28, p < 0.05) as did FA in the midbrain section (R = 0.31, p < 0.02).

**Conclusions:**

This study demonstrates that DTI measures of brain tissue injury can detect injury to a functionally relevant white matter pathway, and that such measures correlate with clinically accepted evaluation indices for INO. The results validate DTI as a useful imaging measure of tissue integrity.

## Introduction

Conventional imaging of the central nervous system (CNS) is well-established for diagnosis of multiple sclerosis (MS) and for testing disease-modifying agents in clinical trials. However, MS lesions that appear similar on conventional imaging display wide heterogeneity on histopathology, with varying degrees of astrogliosis, demyelination and remyelination.

Advanced imaging methods such as diffusion tensor imaging (DTI)[1] offer quantitative measures of tissue injury that promise higher sensitivity to disease than conventional imaging. The measures can also be interpreted in terms of pathological changes such as axonal injury and demyelination.[2] However, the relationship between alterations in tissue architecture and corresponding pathophysiologic signatures is yet to be firmly established. Validating advanced imaging measures as physiologically relevant indices of tissue injury would help justify their use as outcome metrics in therapeutics studies. Towards this end, we studied quantitative imaging and corresponding functional measurements of injury to an eloquent brainstem pathway, the medial longitudinal fasciculus (MLF).

The MLF is a pair of white matter pathways that carries neural signals essential to conjugate eye movements. The MLF connects the paramedian pontine reticular formation (PPRF) and abducens nucleus to the contralateral medial rectus subnucleus of cranial nerve III.[3] When physiologically intact, these systems work together to carry out most of the extraocular eye movements. Injury to the MLF can produce a myriad of ocularmotor as well as otolithic abnormalities.

The most common consequence of lesions in the MLF is internuclear ophthalmoparesis (INO). INO is characterized by slowing of the adducting eye on the side of the MLF lesion[4] and is best appreciated during rapid saccades. INO is one of the most common oculomotor symptoms in MS and can be identified on a clinical exam.

Infrared oculography quantifies the severity of INO to high precision.[3,5] One measure of the severity of INO provided by infrared oculography is the versional disconjugacy index (VDI). VDI is the ratio of the velocity of the abducting to the adducting eye movements. Normally, VDI equals one. The eyes move with similar velocity so that foveation is achieved with stereopsis. With INO, adduction of one eye slows so that VDI increases beyond unity. VDI increases with severity of INO. Infrared oculography is also highly sensitive to injury. Previous work showed that infrared oculography could detect mild cases of INO that a majority of well-trained neurologists missed on a clinical exam.[6]

Difficulty in validation of quantitative imaging results as much from properties of commonly-used scales of functional disability as from the imaging. Commonly-used scales of functional disability such as the EDSS and MSFC are inherently coarse. The precision and sensitivity infrared oculography yield a continuous range of VDI that better matches the quantitative measures provided by DTI.

Furthermore, the functional disability measured by infrared oculography tightly associates with injury to a well-defined neural pathway, the MLF. In contrast, the EDSS and MSFC characterize complex functions, such as walking, that involve neural circuits that are widely distributed and heterogeneous.

DTI provides quantitative measures of tissue injury[7] that reflect axonal integrity and demyelination.[2] In this study, we determine if DTI of the MLF can differentiate patients with INO from control subjects. We also examined the correlation between quantitative DTI measures of tissue injury and quantitative functional measures by infrared oculography.

## Materials and Methods

The institutional review board at University of Texas Southwestern Medical Center reviewed and granted approval for this study. After obtaining written consent, 40 MS patients with chronic INO were examined (26 female, average age 49 ±7 years, range 32–59 years; 26 bilateral INO and 14 unilateral INO; Expanded Disability Status Scale < 6.0 for 17 subjects and, for the remaining 23 subjects, average 6.6, standard deviation of 0.6, range 6.0–7.5) and 15 healthy controls (10 Female, average age 46 ±8 years years, range 28–59 years). Chronic INO was defined as the absence of an episode of diplopia in the past year. All patients had pontine MLF involvement visualized on T2-weighted images. Each subject underwent infrared eye movement evaluation as previously described[3] and MR measurements. As corticosteroid therapy can impact MR measurements,[8]MS patients were free of corticosteroids for at least 3 months prior to the study.

Eye movement disconjugacy measurements were performed by infrared oculography. Differences between left and right eye during left-directed and right-directed 20 degree saccades were characterized using left (left eye abduction velocity divided by right eye adduction velocity) and right (right eye abduction velocity divided by left eye adduction velocity) versional disconjugacy index (L-VDI and R-VDI).[5] Large values of L-VDI correspond to slow adduction of the right eye which we hypothesize to correlate with injury to the right MLF. Similarly, large R-VDI is hypothesized to correlate with injury to the left MLF.

All MR imaging was performed at a whole-body 3T scanner using an 8-channel head coil (Philips Medical Systems, Cleveland, OH). Imaging featured high spatial resolution DTI with physiological triggering to minimize pulsatile artifacts (256x256x20 matrix, 1x1x2 mm voxels, 30 diffusion-weighting gradients with b = 700 sec/mm^2^ and 1 b = 0 volume, NEX = 2, TE = 66 msec, TR = 20 heartbeats). Cardiac triggering used a fingertip plethysmograph to acquire one slice per heartbeat at a delay of 300 msec after the peak of the pulse. Image slices were aligned perpendicular to the posterior edge of the pons and covered the pons. For coregistration purposes, a whole-brain b = 0 image with same angulation as the high spatial resolution DTI was also acquired (256x256x80 matrix, 1x1x2mm voxels, TE = 65 msec, TR = 11688 msec). Images were subject to iterative motion correction[9] in which diffusion gradient directions were updated.[10] The diffusion tensor and scalar measures of tissue integrity ((longitudinal diffusivity [LD, also known as axial diffusivity], transverse diffusivity [TD, also known as radial diffusivity], mean diffusivity [MD] and fractional anisotropy [FA]) were calculated using corrections for partial volume averaging with cerebrospinal fluid.[11]

In addition to the DTI, imaging consisted of a localizer, whole-brain axial T2-weighted image (512x512x28 matrix, 0.45x0.45x4mm voxels, TE = 80msec, TR = 3000msec), axial thin-slice T2-weighted images located at the pons (512x512x32 matrix, 0.45x0.45x2mm voxels, TE = 80msec, TR = 3000msec), sagittal T1 MPRAGE (256x256x160 matrix, 1x1x1mm voxels, TE = 3.7msec, TR = 8.2msec), and axial phase maps (128x128x46 matrix, 2x2x2 mm voxels, TE = 65msec, TR = 11688msec).

**Fig 1:**
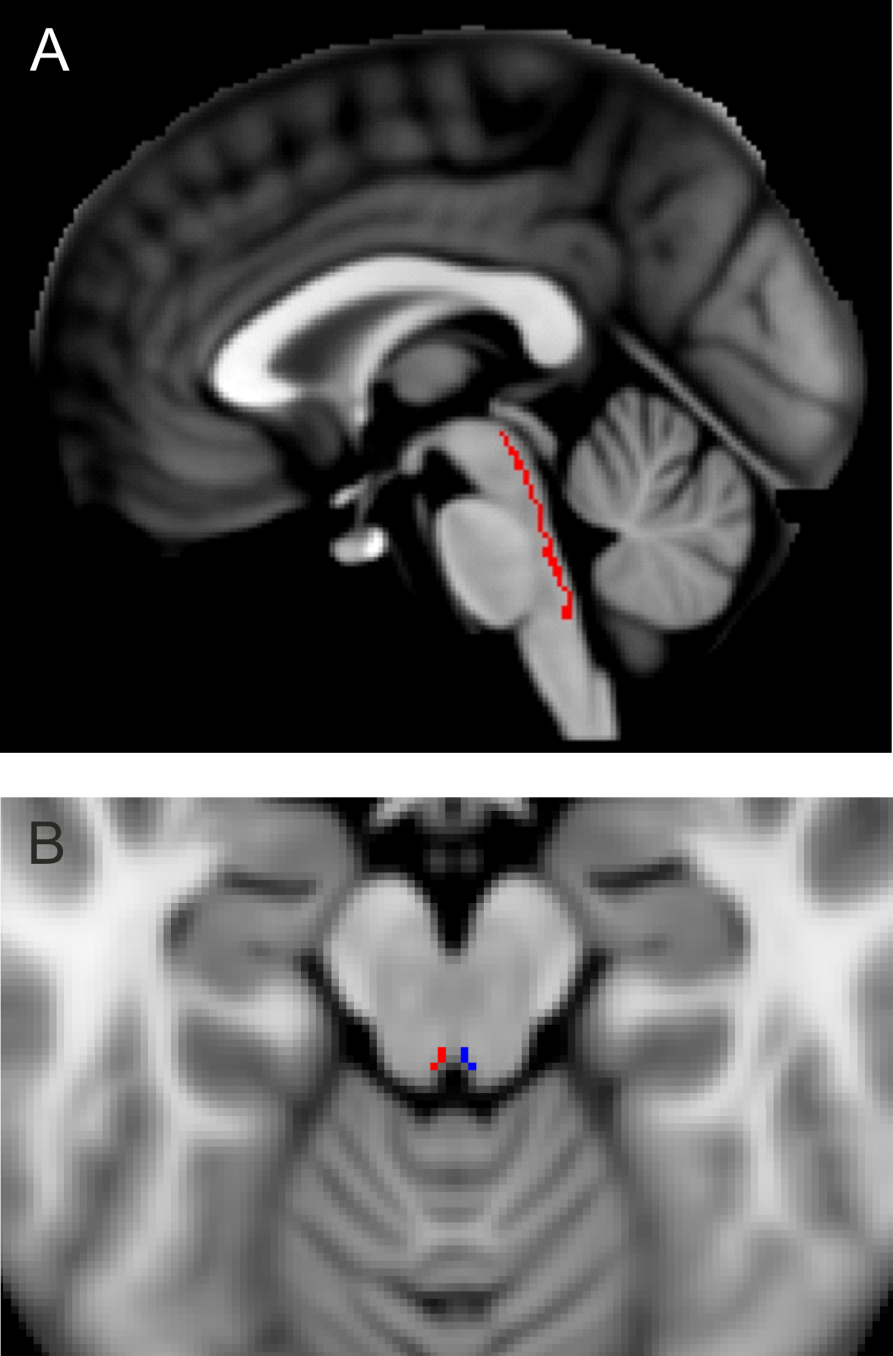
(A) Sagittal View of MLF ROI (red) overlaid on MNI atlas.(B) Axial View of left (blue) and right (red) MLF ROI.

The MLF was localized using an atlas-based method. Regions of interest (ROI) corresponding to the left and right MLF were drawn by a neurologist with expertise in brainstem neuroanatomy (EF) on the MNI atlas (MNI_152_1mm_brain, 152 nonlinear 6^th^ generation symmetric Average Brain Stereotaxic Registration Model)[12] provided by FSL[13] (Fig 1). High spatial resolution DTI b = 0 images covering the pons were then coregistered with the whole-brain b = 0 image, which in turn was coregistered with the MNI atlas using FLIRT[14] and a brainstem mask[15] generated from the Harvard-Oxford subcortical atlas.[16] Transformation matrices were concatenated, inverted and applied to the MLF ROI to transform the ROI to DTI space. ROI placements were then checked manually to ensure alignment and to avoid ventricle and susceptibility artifact. A board-certified radiologist checked the axial thin-slice T2-weighted images to determine if T2 lesions overlapped the MLF. The extent of the lesions was recorded in MNI coordinates by visual comparison between the anatomical images and the MNI atlas. Measures of tissue integrity were determined along the length of the brainstem MLF using the coregistered ROI. The mean within the ROI at each slice location was calculated separately for of LD, TD, MD and FA. The slice levels were then transformed to their corresponding inferior-superior coordinates in MNI space. Values across subjects as a function of inferior-superior coordinates were then fit using penalized splines.[17,18] Because of variability in positioning of the image slices, the ROI was not included in the image in all studies at the inferior and superior extremes. The penalized spline fit was therefore performed only over the range -48mm ≤ z ≤ -8mm in MNI coordinates. A bootstrap test of the equivalence of two curves was then performed to test the null hypothesis that the fits for MS patients were the same as those for controls. DTI measures in the left (right) branches of the MLF with R-VDI (L-VDI) were evaluated using Pearson’s linear correlation coefficient analysis with Bonferroni correction for multiple comparisons. For the correlation analyses, DTI measures from the right and left MLF were considered independently since each MLF contributes independently to the VDI measure.

## Results

**Fig 2:**
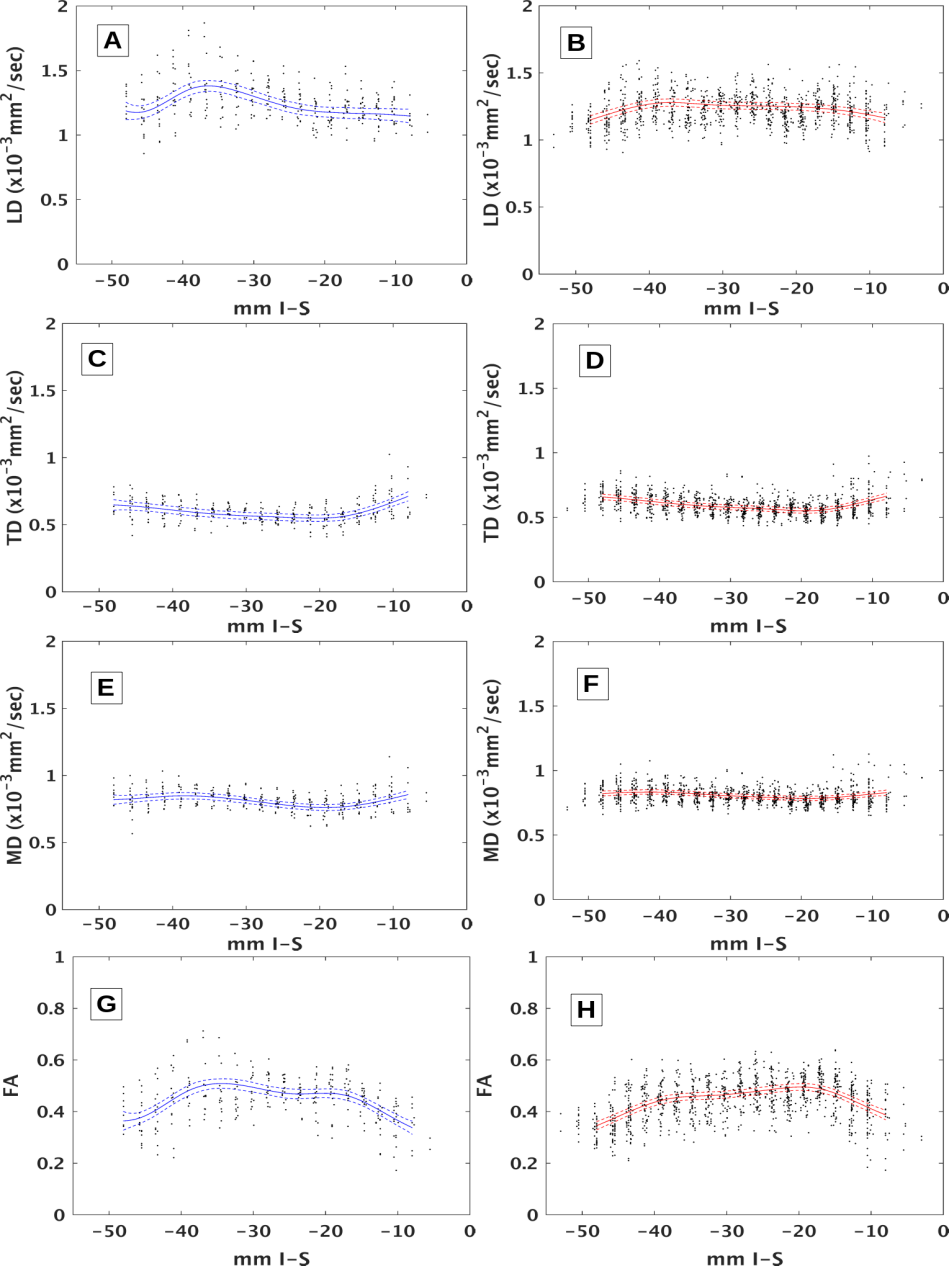
DTI measures with spline fits. Diffusion tensor-derived values at different slice locations (mm in Inferior-Superior direction in MNI space) along with penalized spline fits (solid lines: fit, dashed lines: 95% confidence intervals). Controls are on the left in blue (A,C,E,G), patients on the right in red (B,D,F,H). Parameters are LD (A,B), TD (C,D), MD (E,F) and FA (G,H).

**Fig 3:**
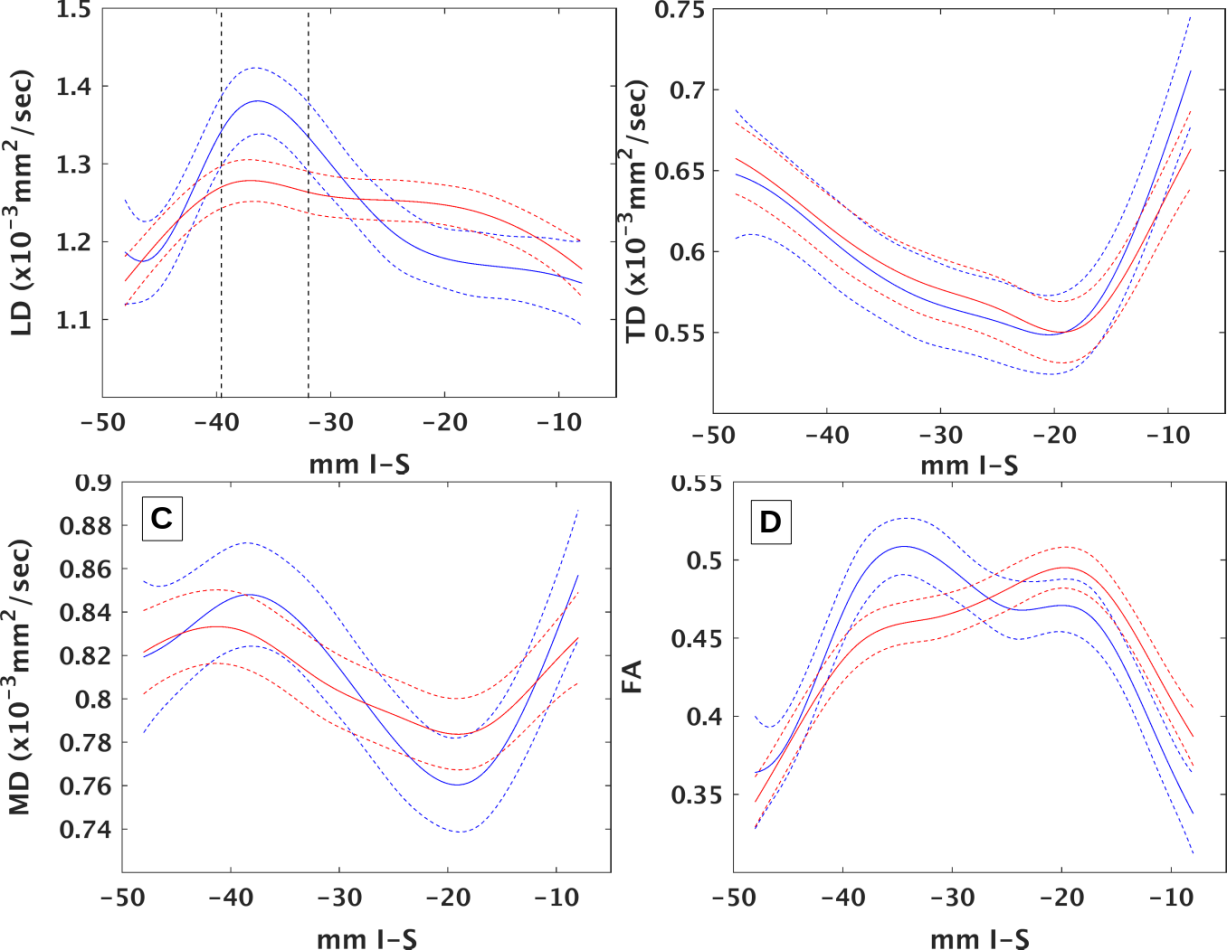
Comparison between patients and controls. Comparisons between controls (blue) and patients (red) of penalized spline fits (solid) and 95% confidence intervals (dashed) of A) LD, B) TD, C) MD and D) FA. Medulla-pons corresponds to coordinates between vertical black dashed lines in A.

**Fig 4:**
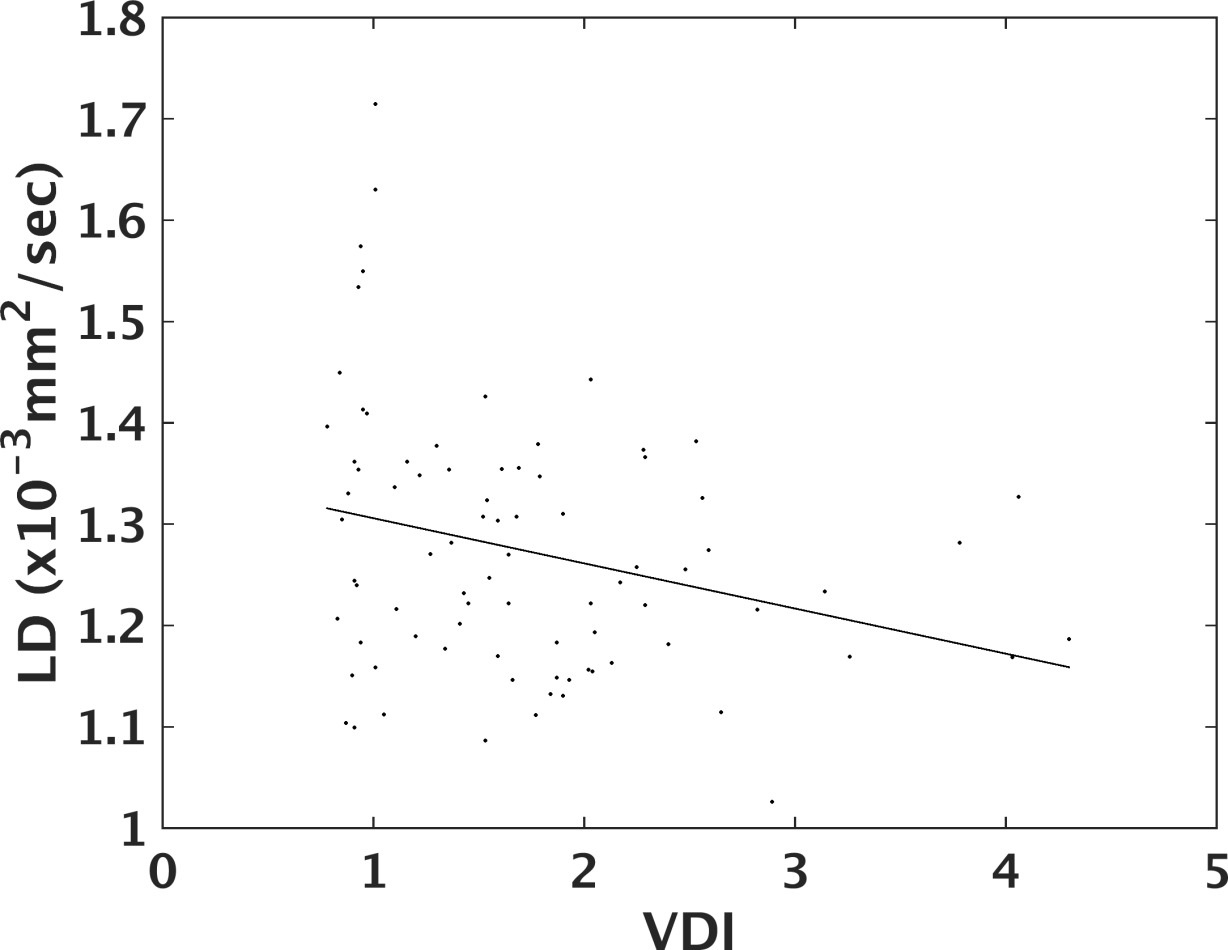
DTI-VDI correlation. Correlation between VDI measures and LD in the medulla-pons region along with a regression line.

DTI of the MLF differentiates MS from controls in certain sections of brainstem. Fig 2 shows penalized spline fits with 95% confidence intervals for tensor measures of tissue integrity for MS patients and controls. The curves for MS patients and controls are significantly different for LD (p < 0.03) and FA (p < 0.0004) but not for TD or MD (p > 0.1). The differences between curves become more evident in Fig 3, which overlaps curves for MS patients and controls. The confidence intervals for LD do not overlap in two regions, indicating significant differences. The first, medulla-pons, region ranges from upper medulla up through mid-pons (-39 ≤ z ≤-32). The second, midbrain, region ranges from the transition between the pons and midbrain up through the level of the red nuclei (-21 ≤ z ≤ -18). Confidence regions for FA do not overlap in similar regions (-39 ≤ z ≤ -31 and -15 ≤ z ≤ -8). Confidence intervals overlap over the entire length of the brainstem for TD and MD, indicating that the differences seen in FA are largely driven by differences in LD. DTI correlates with quantitative infrared oculography. Fig 4 compares the mean values for LD across all ROIs in the medulla-pons region with their corresponding VDI on the same side. Pearson’s linear correlation coefficient analysis shows significant correlation (R = -0.28, p < 0.05). Correlation between LD and VDI in the midbrain was not significant. Correlation between FA and VDI was not significant in either region.

T2 lesions overlapped the MLF ROI in 33 of the 40 patients. The lesions were in the medulla-pons region (-39 ≤ z ≤-32) in 11 patients, in the midbrain region (-21 ≤ z ≤ -18) in 5 patients and in both regions in 1 patient. To assess the impact of T2 lesions on DTI measures, values within and outside T2 lesions were compared. For each patient and for each region (medulla-pons or midbrain), the MLF ROI was split into two parts—within the T2 lesion and outside the T2 lesion—and paired by patient. The mean value of each DTI measure (LD, TD, FA or MD) was taken in each part. A paired t-test comparing within- and outside-lesion DTI measures found no significant differences (p > 0.7). Data from the patient without a T2 lesion was excluded from the analysis. If a T2 lesion did not reside in a region for a given patient, data was not included in the comparison.

## Discussion

The work presented here demonstrates that injury to a specific brainstem pathway, the MLF, can be detected by DTI. DTI measures of tissue integrity also correlate with quantitative measures of functional impairment. This further expands the findings of our previous pilot study using a smaller cohort and 1.5T imaging.[19]

LD is often interpreted as a marker of axonal integrity.[2] Reduced LD in MS patients in the medulla-pons region aligns with this interpretation. After axonal transection, the formation of obstacles to water diffusion is expected. In MS, formation of ovoids and proliferation of glial cells are known phenomena on histology [20] and may contribute to decreased diffusivity. Decreased efficiency of axonal conduction is also expected after transection, which would explain concomitant increase in VDI. Correlation with quantitative infrared oculography further suggests that the measured change in DTI in this specific region has a corresponding significant, measurable, and relevant effect on physiologic function of that tissue region.

As MS is a demyelinating disease, we expected TD, often considered a marker for demyelination, to be higher in MS patients than in controls. However, we did not observe a difference in TD between MS and controls. The most straightforward interpretation of this result is that the imaging approach does not have sufficient sensitivity to demyelination in this region. Higher signal to noise ratio, larger sample size or different approaches to quantifying myelin density, such as magnetization transfer, may be required to quantify demyelination in the MLF.

The observation that LD is higher among MS patients than in controls in the midbrain region (-21 ≤ z ≤ -18) is harder to interpret. Elevated diffusivity can be an artifact associated with partial volume averaging with nearby cerebrospinal fluid. However, ROIs were placed to avoid overlap with CSF spaces and corrections were used to account for partial volume averaging.[11] Furthermore, partial volume averaging is expected to result in elevation in both TD and MD, which was not observed. A group comparison was performed to check the results. Differences between patients and controls when taking isolated segments were significant as per a t-test in the medulla-pons (p < 4 x 10^−5^). After the acute stage of lesion formation, LD is typically increased in MS patients, but these observations are not performed in the brainstem. There is increasing recognition that the interpretation of DTI indices in terms of injury, such as demyelination and axonal transection, is incomplete. For example, low FA is typically interpreted as an indicator of tissue injury, but elevated FA has been associated with injury due to Alzheimer’s disease [21]. Such unexpected behavior of FA has been attributed to complexity of fiber architecture, such as fiber crossings. We do not expect fiber crossings in the regions examined in this study, but other factors such as fiber tortuosity, density and fanning can affect DTI indices [22,23]. The results suggest that DTI can detect differences between patients and controls in the medulla-pons region and in the midbrain, but that these differences may relate to injury, differences in architecture or a combination. Such ambiguity underscores the limitations of DTI. More sophisticated approaches [24] may be needed to accurately assess injury in this region of the brain.

Measuring DTI characteristics of the MLF is challenging. In principle the MLF may be identified by deterministic or probabilistic tractography, but our previous studies found that the small diameter of the MLF (each tract consisting of only approximately 10,000 nerve fibers) and the presence of large, parallel pathways make such approaches inconsistent and unreliable.[25] We therefore used a simple atlas-based approach to identify the MLF. It may be possible to improve results by using a more sophisticated atlas.[26,27] However, a problem with any atlas-based approach is that the atlas is typically generated using histology from a single normal subject, not one with the demyelinating lesions and atrophy seen in multiple sclerosis. A more accurate atlas may therefore not result in more accurate identification of the tissue of interest. It is possible to identify the MLF directly by anatomical imaging,[28] particularly at 7T.[25] However, the contrast between MLF and its surrounding regions often disappears in MS patients [29]. Robust methods for identifying small but functionally important pathways, and accounting for the low signal to noise ratio in these pathways, are important goals for future work. Nonetheless, errors in ROI placement might be expected to have a minimal effect on our results, since the injury to adjacent fiber tracts would be expected to be similar to the injury sustained by the MLF.

Mean values of LD, TD, MD and FA were taken at each slice level of each ROI in each subject. An alternative approach would be to include values for all voxels without taking the mean. Including all voxels in the analysis is feasible but introduces complexity. The transformation of the ROI from the MNI template to each subject’s image coordinates does not preserve the number of voxels at each slice level. Therefore, the number of voxels would have to be included as a covariate in the analysis. Due to the limitations of the statistical power of the measurement, a number of questions remain unanswered. Right and left MLF were not considered separately. It would be interesting to determine the extent to which the observed changes in DTI are due to tissue in lesions as opposed to normal appearing white matter. A clean test would include additional patients who do not have lesion involvement in the MLF—a cohort not included in this study. With a more extensive study, it may be possible to investigate differences between subtypes of INO, such as anterior vs. posterior or unilateral vs. bilateral.

## Conclusion

This study is a step toward validation of quantitative measures from advanced imaging in a patient-specific model. This study demonstrates that DTI can detect injury to a small brainstem pathway, the MLF. Furthermore, DTI measures of injury correlate with a precise and sensitive measure of functional disability, infrared oculography, in the presence of INO. Studies focusing on neural systems with well-defined function provide a patient-relevant approach for testing quantitative imaging.
